# Adsorption–desorption characteristics of coal-bearing shale gas under three-dimensional stress state studied by low field nuclear magnetic resonance spectrum experiments

**DOI:** 10.1038/s41598-024-54532-9

**Published:** 2024-03-06

**Authors:** Hunan Tian, Jupeng Tang, Shipeng Zhang, Xin Zhang

**Affiliations:** 1 School of Energy and Chemical Engineering, Xinjiang Institute of Technology, Aksu, 843100 China; 2 School of Mechanics and Engineering, Liaoning Technical University, Fuxin, 123000 Liaoning China

**Keywords:** Coal-bearing shale, Nuclear magnetic resonance *T*_2_ spectrum, Average effective stress, Adsorption–desorption, Fossil fuels, Coal

## Abstract

The micro-scale gas adsorption–desorption characteristics determine the macro-scale gas transport and production behavior. To reveal the three-dimensional stress state-induced gas adsorption–desorption characteristics in coal-bearing shale reservoirs from a micro-scale perspective, the coal-bearing shale samples from the Dongbaowei Coal Mine in the Shuangyashan Basin were chosen as the research subject. Isothermal adsorption–desorption experiments under three-dimensional stress state were conducted using the low field nuclear magnetic resonance (L-NMR)* T*_2_ spectrum method to simulate the in-situ coal-bearing shale gas adsorption–desorption process. The average effective stress was used as the equivalent stress indicator for coal-bearing shale, and the integral of nuclear magnetic resonance *T*_2_ spectrum amplitude was employed as the gas characterization indicator for coal-bearing shale. A quantitative analysis was performed to examine the relationship between gas adsorption in coal-bearing shale and the average effective stress. And a quantitative analysis was performed to examine the relationship between the macroscopic and microscopic gas quantities of coal-bearing shale. Experimental findings: (1) The adsorption–desorption process of coal-bearing shale gas follows the L-F function model and the D-A-d function model respectively with respect to the amount of gas and the average effective stress. (2) There is a logarithmic relationship between the macroscopic and microscopic gas quantities of coal-bearing shale during the adsorption–desorption process. This quantitatively characterizes the differences in the curves, which may be related to the elastic–plastic deformation, damage and fracture of the micropores in coal-bearing shale, as well as the hysteresis of gas desorption and the stress field of the gas occurrence state.

## Introduction

With the successful exploration and development of unconventional natural gas resources such as coalbed methane, sandstone gas, and shale gas, the commercial value of coal-bearing shale gas resources with abundant reserves and wide distribution has become increasingly evident. As an unconventional natural gas resource, coal-bearing shale gas has significant differences from marine or transitional shale gas in terms of organic matter abundance, type, hydrocarbon generation, hydrocarbon release characteristics, and preservation conditions^1^. Currently, there is an urgent need to explore the adsorption and desorption laws of coal-bearing shale gas to provide relevant theories and experimental references for the comprehensive development of deep coal-bearing shale gas resources in the future.

Scholars have conducted numerous fruitful studies on the adsorption and desorption laws of shale gas. Bhowmik et al.^2^ believe that the shale adsorption capacity is mainly related to the type and maturity of organic matter. Ross and Bustin^3^ suggests that shale adsorption capacity is associated with factors such as pore structure distribution, kerogen type, and clay content. Yang et al. ^4^ investigated the influence of pressure, temperature, and particle size on shale gas adsorption and desorption. Tang et al.^5^ found through on-site desorption experiments with shale that shale gas desorption ability is closely related to organic matter content. Zhou et al. ^6^ found that the nuclear magnetic resonance transverse relaxation time spectrum of shale methane adsorption process exhibits three distinct peaks. Zhao et al. ^7^ studied the isothermal desorption curves of shale methane and divided them into three stages: rapid desorption, slow desorption, and inefficient desorption. They also found that the inefficient desorption stage contributes the most to shale gas production. Chen et al. ^8^, through molecular simulation studies, discovered that with increasing temperature and pressure, the adsorption capacity of shale pores for methane molecules increases. The adsorption behavior of methane in shale pores may be related to electrostatic energy and van der Waals forces. Maryam et al.^9^, through molecular dynamics numerical simulations of shale methane adsorption, found that within the methane pressure range of 0–3 MPa, the adsorption capacity decreases with increasing temperature, and the adsorption process conforms to the Langmuir isotherm model. Lin et al.^10^ studied the effects of total organic carbon content (TOC), organic matter type, organic matter maturity, minerals and clay minerals, water content, and pore characteristics on methane adsorption behavior in shale. They also found that the characteristics of gas adsorption behavior under high temperature and high pressure conditions indicate that the adsorption behavior is difficult to describe using the Langmuir equation. Bai et al.^11^, through experimental research, discovered that there are differences between the theoretical adsorption capacity and the actual adsorption capacity of methane in shale. The actual adsorption capacity of shale for methane is related to methane adsorption capacity, shape factor, and connectivity of matrix pore structure. Bumb et al.^12^ discovered that shale adsorption–desorption follows the Langmuir isotherm adsorption theory. Chareonsuppanimit et al.^13^ and Soulaine et al.^14^ further provided shale isothermal adsorption SLD model and desorption micro-continuum model. Ekundayo and Rezaee^15^ and Chen et al.^16^ discovered that shale desorption exhibits hysteresis and this phenomenon may be related to variations in shale pore throat size. Li et al.^17^ comprehensively considered factors such as water content, temperature, pressure, and organic matter content that affect shale adsorption law. Lin et al.^18^ found that shale desorption process is multi-stage, and the rapid desorption stage has the greatest impact on shale gas production. Zhou et al.^19^ believed that both monolayer adsorption and micropore filling exist in the shale adsorption process. Wang et al.^20^ proposed an isothermal adsorption model for shale gas considering excess adsorption capacity. Dong et al.^21^ developed a DR-LFρ model for high-pressure isothermal adsorption of shale based on adsorption phase density theory. Yao et al.^22,23^ conducted methane adsorption experiments on coal powder and shale powder and found that the integral of nuclear magnetic resonance *T*_2_ spectra amplitude can quantitatively characterize the corresponding microscale adsorption state, free state, and bulk gas content during the adsorption process. Tang et al.^24,25^ studied the hysteresis effect of adsorption–desorption of micro adsorbed and porous medium- confined gas in coal-bearing shale using L-NMR *T*_2_ relaxation spectrum amplitude integration quantitative method.

Many previous theories and experiments have focused on the study of the macroscopic adsorption–desorption laws of shale gas and their influencing factors. In these experiments, only adsorption pressure (pore pressure) is considered, while actual coal-bearing shale gas extraction from shale formations involves the combined effects of overlying strata pressure, surrounding rock pressure, and reservoir pore-fracture fluid pressure. Therefore, the conclusions obtained from these experiments have certain limitations in guiding coal-bearing shale gas extraction under three-dimensional stress state. As gas in coal-bearing shale formations has abundant reserves, its in-situ state of occurrence is influenced by the three-dimensional stress field, which cannot be ignored in the study of its adsorption–desorption characteristics. The micro-scale adsorption–desorption characteristics of gas determine its macroscopic generation and migration behavior. Therefore, it is necessary to conduct experimental research on the adsorption–desorption characteristics of coal-bearing shale gas under three-dimensional stress state based on previous studies. By using nuclear magnetic resonance spectrum, the adsorption–desorption laws of coal-bearing shale gas can be explored from a microscopic perspective, providing theoretical references for coal-bearing shale gas extraction.

This article focuses on the research of the coal-bearing shale in the 36# coal-bearing floor of the third mining area of Dongbaowei Coal Mine in the Shuangyashan Basin, China. It conducts experimental and theoretical research on the adsorption–desorption behavior of coal-bearing shale gas under three-dimensional stress state using L-NMR *T*_2_ spectrum method. The new findings have certain reference significance for the safe and efficient exploitation of coal-bearing shale gas resources in the Shuangyashan Basin.

## Experimental principle and process

### Experimental principle

L-NMR has been widely used as a new fast, non-destructive online detection technology in the characterization of coal-bearing shale pore structures, permeability, adsorption–desorption properties, and enhancement of shale gas recovery rates^26^. L-NMR technique detects the nuclei in methane, and the signal amplitude is proportional to the mass of methane within the detection range. Therefore, the *T*_2_ spectrum amplitude integration can be used to characterize the gas content of coal-bearing shale fractures^22,24^. The relationship between the transverse relaxation time *T*_2_ and the coal-bearing shale fractures is shown by Eq. (1).

1 $$\frac{1}{{T_{2} }} \approx F_{s} \frac{{\rho_{2} }}{r}$$

In the equation below, $F$ represents the geometric shape factor, dimensionless;$r$ average pore radius, μm; $\rho_{2}$ is the surface relaxation strength, μm/ms; *T*_2_ is the gas transverse relaxation time, ms.To quantitatively study the adsorption–desorption characteristics of coal-bearing shale gas under three-dimensional stress state, we introduce a parameter that can reflect the overall force situation of coal-bearing shale gas samples under three-dimensional stress state the average effective stress $\sigma_{e}$^27^, as shown in Eq. (2).

2 $$\sigma_{e} = \frac{1}{3}(\sigma_{1} + 2\sigma_{2} ) - \frac{1}{2}(P_{1} - P_{2} )$$

In Eq. (2),$\sigma_{e}$ represents the average effective stress in MPa;$\sigma_{1}$ represents the axial pressure in MPa;$\sigma_{2}$ represents the confining pressure in MPa;$P_{1}$ represents the initial pore pressure in MPa;$P_{2}$ represents the equilibrium pore pressure in MPa.

### Sample collection and preparation

Due to the limitations of the laboratory's three-axis nuclear magnetic resonance core holder, standard specimens with a diameter of 25 mm and a length of 50 mm were used for the experimental rock cores. Small-sized original rock samples have a low adsorption capacity under low adsorption pressure conditions, resulting in very weak corresponding nuclear magnetic resonance signals. Additionally, the nuclear magnetic resonance spectrometer has a certain background signal "noise." Under low adsorption pressure conditions, methane test signals may be partially obscured by the background signal "noise." Furthermore, the internal natural cracks in coal-bearing shale vary randomly, leading to significant differences in test results. Therefore, in this study, we attempted to use small-sized standard shale samples with relatively large porosity and adsorption capacity (diameter 25 mm, length 50 mm) as approximations to the original shale for experiments to reduce experimental errors.

**Figure 1:**
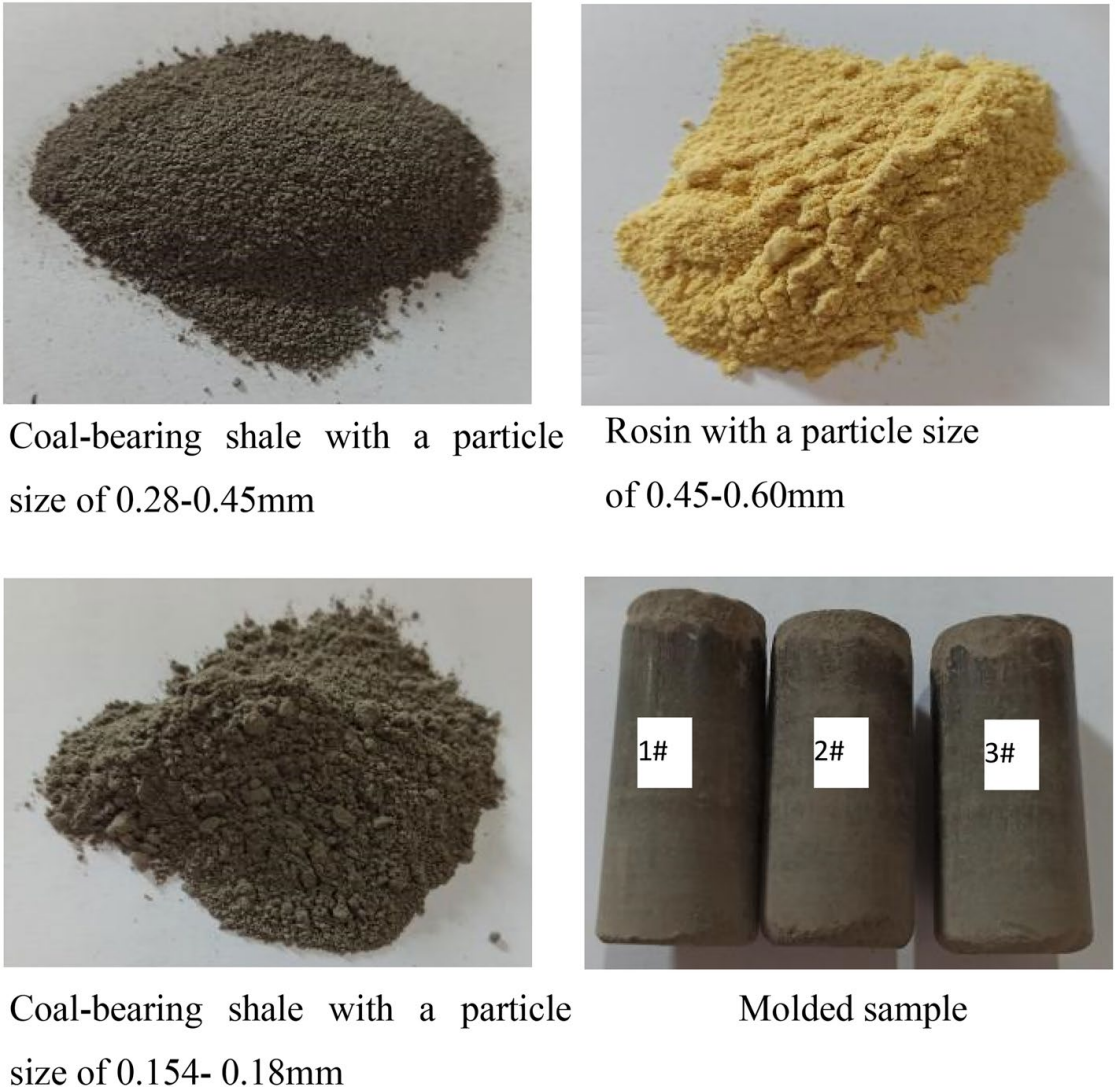
Coal-bearing shale sample.

The experimental samples were from the unweathered shale of the 36 # coal seam floor in the third mining area of Dongbao Coal Mine in the Shuangyashan Basin,China.Its physical parameters were measured as follows: bulk density of 2.43 g/cm^3^, porosity ranging from 1.6 to 5.0%, compressive strength from 3.0 to 18.7 MPa, and elastic modulus of 13.5 GPa. A sufficient amount of rock powder was taken, dried for 24 h, and then cooled. The experimental samples were prepared as shown in Fig. 1 and their parameters were listed in Table 1, using a 200t pressure testing machine.

**Table 1:** Characteristic parameters of samples.

Sample	Particle size		Adhesive	Diameter/mm	Height/mm
Shale	0.28–0.45 mm	0.154–0.18 mm	Rosin		
1#	18.70 g	37.45 g	3.15 g	50.12	24.87
2#	18.60 g	38.54 g	3.10 g	50.05	25.12
3#	17.62 g	37.66 g	3.12 g	50.00	24.93

### Experimental apparatus

**Figure 2:**
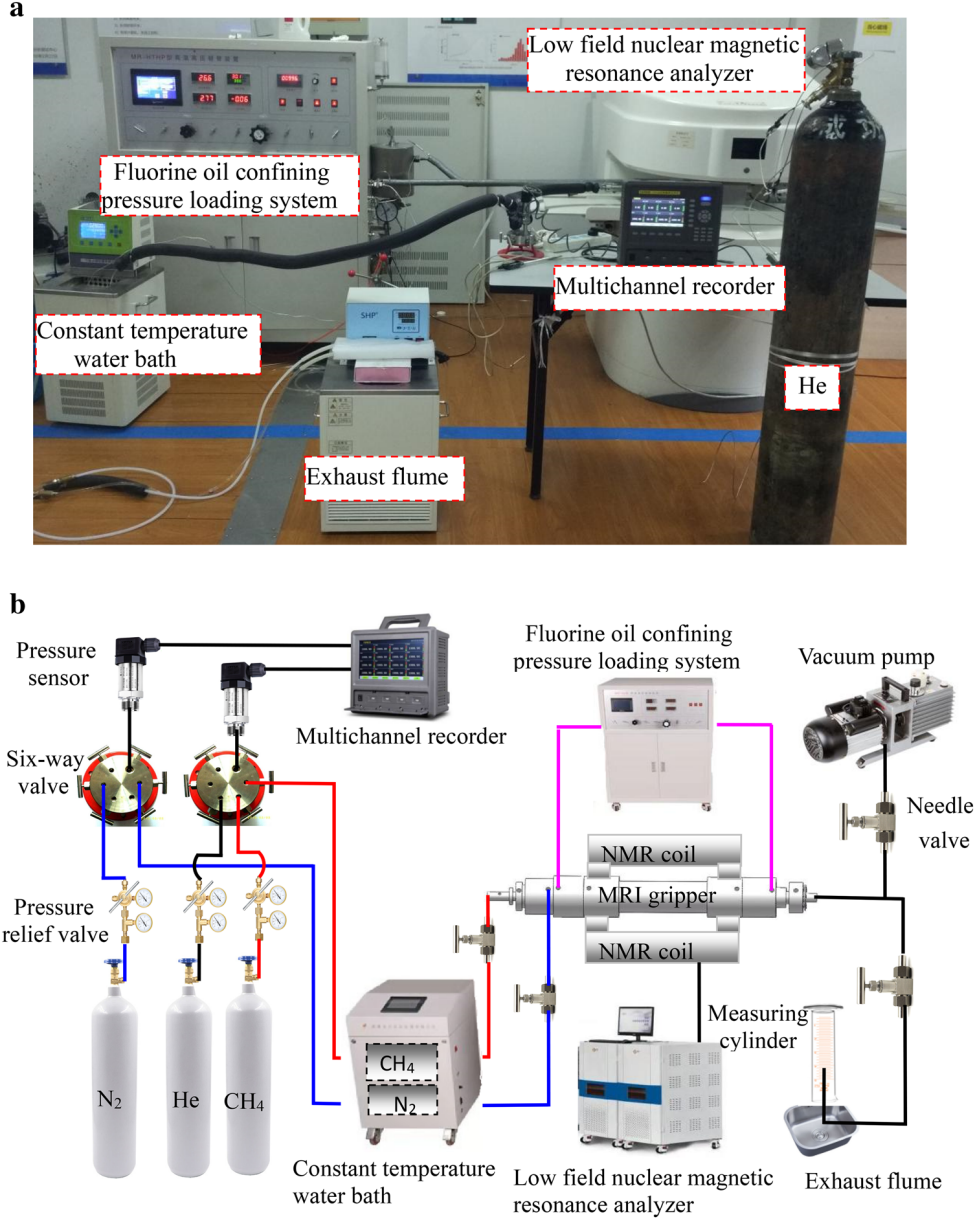
(**a**) Field experiment on coal-bearing shale gas adsorption–desorption characteristics under the three-dimensional stress state. (**b**) Connection diagram of NMR experimental device for gas adsorption–desorption characteristics of coal-bearing shale under the three-dimensional stress state.

The experimental apparatus for gas adsorption–desorption characteristics of coal-bearing shale under three-dimensional stress state, as shown in Fig. 2, mainly consists of a data acquisition preprocessing system, L-NMR data acquisition and analysis system, constant-temperature water bath system, and fluorine oil confining pressure loading system. The experiments were conducted at the Suzhou Numag Analytical Instrument Co. Ltd. testing center using a MacroMR12-150H-1 nuclear magnetic resonance analyzer with a coil diameter of 70 mm. High-purity methane was used as the adsorption gas, and nitrogen was used as the driving gas. The testing environment temperature was maintained at 25 ± 0.5 ℃.

### Experimental procedure

The coal-bearing shale at a depth of 578 m in the East Baowei Coal Mine, Duanyashan Basin, is considered for the experiments. To ensure safety in experimental operations and equipment usage, a method of applying in-situ stress and gas pressure without reduction was used in this location^28^. The confining pressure coefficient was set to 1.2, the vertical stress coefficient was set to 4.0, the pore pressure coefficient was set to 1.0, and the average bulk density of the overlying strata was taken as 25 kN/m^3^ ^29^. The laboratory designed simulations at burial depths of 350 m, 550 m, 750 m, 850 m, 1050 m, 1200 m, and 1500 m.

Coal-bearing shale gas mainly exists in the form of adsorbed gas, porous medium-confined gas, and a small amount of matrix solution state^25^. To eliminate interference from trace free gas signals in the nuclear magnetic resonance spectra of coal-bearing shale gas during the experiments due to the clamping heads of the nuclear magnetic resonance holder and the “space” trace free gas in the rock core samples, it is necessary to understand the characteristics of free gas nuclear magnetic resonance spectra. According to the research findings of Tang et al.^24^, it is known that the nuclear magnetic resonance spectrum curve of free gas has only one characteristic peak, and the nuclear magnetic resonance *T*_2_ spectrum ranges from 93.00 to 2710.63 ms. The specific calibration experiments are not elaborated here.

Coal-bearing shale samples were placed in the three-axis nuclear magnetic resonance holder, and the experimental apparatus was connected as shown in Fig. 2. The experiments were conducted following the “Shale Methane Isothermal Adsorption Test Method” (GB/T35210.1-2017)^30^ and the “High-Pressure Isothermal Adsorption Test Method for Coal” (GB/T19560-2008) ^31^ to study the characteristics of coal-bearing shale gas in the adsorption–desorption process under the three-dimensional stress state.

#### Isothermal adsorption experiment

Start the L-NMR analyzer at least 48 h in advance to stabilize the magnet temperature at 32 ± 0.01 ℃, ensuring the magnetic field stability during the subsequent testing process.

1. Evacuate the system by purging with high-purity helium gas. Maintain a vacuum pump negative pressure of 10 kPa and continuously evacuate for 120 min to minimize moisture and air impurities in the experimental system.
2. Apply axial pressure and open the nitrogen cylinder. Load axial pressures sequentially at 2.24 MPa, 3.61 MPa, 4.47 MPa, 5.43 MPa, 6.44 MPa, 7.45 MPa, and 9.34 MPa.
3. Apply confining pressure and temperature control. Start the high-pressure fluorinated oil temperature control circulation system (circulating fluorinated oil temperature at 25 ± 0.5 ℃). Apply confining pressures sequentially at 1.78 MPa, 2.97 MPa, 4.04 MPa, 5.02 MPa, 6.02 MPa, 7.01 MPa, and 8.79 MPa using the fluorinated oil.
4. Measure the free space volume. Introduce 99.99% high-purity helium gas at the pore pressure inlet and measure the free space volumes of the experimental chamber (nuclear magnetic resonance holder cavity) and connected pipeline valves, etc.
5. Evacuate the system. Remove helium gas from the system at the pore pressure outlet, close the pore pressure outlet, and continuously evacuate at a vacuum pump negative pressure of 10 kPa for 120 min.
6. Acquire baseline signals. After evacuating, acquire baseline signals of the coal shale sample using the L-NMR analyzer.
7. Apply pore pressure. Close the outlet of the gas reference cylinder in the constant-temperature water bath and connect the gas cylinder (containing 99.99% methane) via a methane pressure reducing valve to apply initial pore pressures sequentially at 0.59 MPa, 1.00 MPa, 1.45 MPa, 1.78 MPa, 2.41 MPa, 2.74 MPa, and 3.48 MPa. After disconnecting the gas cylinder, close the inlet of the reference cylinder. After the reference cylinder is stabilized and heated in the constant-temperature water bath, open the inlet of the reference cylinder and apply pore pressure to the sample through the three-axis non-magnetic nuclear magnetic resonance holder. The equilibrium pore pressures correspond to 0.48 MPa, 0.82 MPa, 1.28 MPa, 1.74 MPa, 2.35 MPa, 2.70 MPa, and 3.41 MPa, respectively.
8. Data acquisition and recording. During the adsorption experiment, do not remove the sample and sequentially acquire and record data for each adsorption equilibrium point.

#### Isothermal desorption experiment

Immediately after completing the isothermal adsorption nuclear magnetic resonance spectrum experiment for coal shale gas under three-dimensional stress state, proceed to the isothermal desorption nuclear magnetic resonance spectrum experiment using the pressure reduction desorption method. The specific procedure is as follows:

1. Release pore pressure. Release pore pressures sequentially at 3.48 MPa, 2.95 MPa, 2.47 MPa, 1.97 MPa, 1.49 MPa, 1.05 MPa, 0.61 MPa, and 0.25 MPa, corresponding to equilibrium pore pressures of 3.41 MPa, 3.01 MPa, 2.48 MPa, 1.98 MPa, 1.51 MPa, 1.06 MPa, 0.63 MPa, and 0.49 MPa.
2. Release confining pressure. Release confining pressures sequentially at 8.79 MPa, 7.49 MPa, 6.54 MPa, 5.47 MPa, 4.52 MPa, 3.51 MPa, 2.49 MPa, and 0.95 MPa.
3. Release axial pressure. Release axial pressures sequentially at 9.34 MPa, 8.18 MPa, 7.23 MPa, 6.24 MPa, 5.15 MPa, 4.16 MPa, 3.25 MPa, and 1.77 MPa.
4. Data acquisition and recording. Sequentially acquire and record data for each desorption equilibrium point.

The results of three sets of nuclear magnetic resonance spectrum experiments on the adsorption–desorption characteristics of coal-bearing shale gas under three-dimensional stress state were consistent. Here, we provide the results of the 1# coal-bearing shale sample.

## Experimental results analysis

With reference to the methods for characterizing adsorption and desorption quantities of coal and shale, we define the cumulative adsorption of coal-bearing shale gas under three-dimensional stress state as the gas content of coal-bearing shale gas under the corresponding equilibrium pressure conditions. The desorption quantity of coal-bearing shale gas under three-dimensional stress state is equal to the difference between the maximum cumulative adsorption of coal-bearing shale gas during the adsorption process and the gas content of coal-bearing shale gas during the desorption process. The analysis of the adsorption–desorption characteristics of coal-bearing shale gas under three-dimensional stress state in nuclear magnetic resonance spectra is as follows.

### Adsorption–desorption experimental results of coal-bearing shale gas

From the experimental scheme and Eq. (2) for adsorption–desorption nuclear magnetic resonance spectra under the three-dimensional stress state in coal-bearing shale gas, we obtain the following average effective stresses during the adsorption process: 1.40 MPa, 2.27 MPa, 2.82 MPa, 3.40 MPa, 3.78 MPa, 4.43 MPa, and 5.53 MPa. The average effective stresses during the desorption process are: 5.53 MPa, 4.74 MPa, 4.29 MPa, 3.76 MPa, 3.23 MPa, 2.67 MPa, 2.12 MPa, and 0.85 MPa.

**Figure 3:**
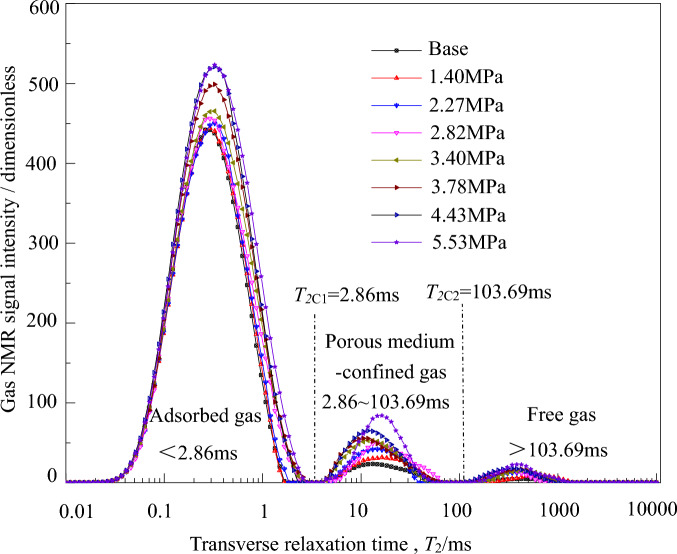
*T*_*2*_ spectrum of coal-bearing shale gas in adsorption process.

**Figure 4:**
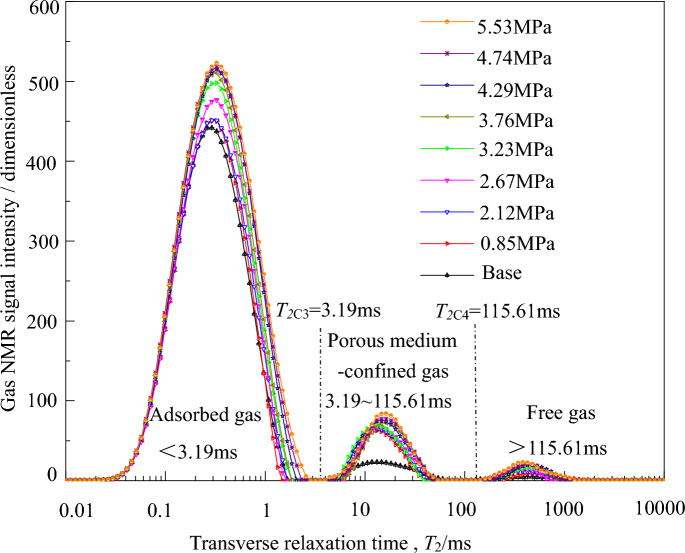
*T*_*2*_ spectrum of coal-beraing shale gas in desorption process.

The results of the adsorption–desorption characteristics of coal-bearing shale gas under the three-dimensional stress state in nuclear magnetic resonance spectra are shown in Figs. 3 and 4. The nuclear magnetic resonance *T*_2_ spectrum curve exhibits three distinct characteristic peaks and two different *T*_2_ cutoff values, consistent with the conclusions of Tang Jupeng et al.^24^ and Yao et al.^32^.

From Fig. 3, it can be seen that as the adsorption equilibrium pressure (average effective stress) continues to increase, the adsorption of coal-beraing shale gas increases, and the corresponding nuclear magnetic resonance *T*_2_ spectrum signal strength also increases. From Fig. 4, it can be observed that as the desorption equilibrium pressure (average effective stress) decreases, the desorption of coal-bearing shale gas increases, and the corresponding nuclear magnetic resonance *T*_2_ spectrum signal strength decreases.

According to the research conclusions of Tang Jupeng et al.^24^, the *T*_2_ spectrum range of free gas is from 93.00 to 2710.63 ms. In Fig. 3, the part where *T*_2_ is greater than 103.69 ms during the adsorption process is the *T*_2_ spectrum of free gas, while the other two parts are the *T*_2_ spectra of porous medium-confined gas and adsorbed gas. According to Eq. (1), the smaller the pore fracture radius size, the smaller the *T*_2_ value of coal-bearing shale gas. Therefore, the range in Fig. 3 where *T*_2_ is less than 2.86 ms corresponds to the *T*_2_ spectrum of adsorbed gas, and the last part is the *T*_2_ spectrum of porous medium-confined gas (2.86–103.69 ms). Similarly, during the desorption process in Fig. 4, the *T*_2_ spectrum of free gas (> 115.61 ms), the *T*_2_ spectrum of adsorbed gas (< 3.19 ms), and the *T*_2_ spectrum of porous medium-confined gas (3.19–115.61 ms) can be identified.

### Adsorption–desorption characteristics of adsorbed gas/porous medium- confined gas

Using average effective stress as the equivalent stress indicator for coal-bearing shale and the integral of nuclear magnetic resonance *T*_2_ spectrum amplitude as the quantitative characterization indicator for adsorbed gas /porous medium-confined gas in coal-bearing shale. The micro-adsorbed/micro-porous medium-confined gas-related experimental characteristics during the adsorption–desorption process of coal-bearing shale gas under three-dimensional stress state are analyzed as follows.

#### Integral of *T*_2_ spectrum amplitude of adsorbed gas conforms to D-R and Weibull functions with average effective stress

1. Preferred adsorption–desorption model.

The quantity of adsorbed gas in coal-bearing shale can be quantitatively characterized using the integral of micro-adsorbed gas nuclear magnetic resonance *T*_2_ spectrum amplitude ^24^. In the adsorption–desorption process of coal-bearing shale gas under three-dimensional stress state, there is no established function representation model for micro-adsorbed gas. Therefore, commonly used adsorption–desorption models are selected to fit the relationship between the quantity of adsorbed gas (integral of adsorbed gas *T*_2_ spectrum amplitude) and the average effective stress. The model with the maximum coefficient of determination *R*_2_ is chosen as the adsorption–desorption model for micro-adsorbed gas. The expressions of the relevant models and the fitting results are shown in Tables 2 and 3.

**Table 2:** Optimization of adsorbed gas model in adsorption.

Model	Model expression	Adsorption process *R*^*2*^
L	$V = \frac{{PV_{L} }}{{P_{L} + P}}$	0.95865
B-BET	$V = \frac{{V_{m} CP}}{{\left( {P^{0} - P} \right)\left[ {1 + \left( {C - 1} \right)\left( {P/P^{0} } \right)} \right]}}$	0.90802
L-F	$V = \frac{{K_{b} P^{m} V_{L} }}{{1 + K_{b} P^{m} }}$	0.99609
D-P	$V = kP + \frac{{PV_{L} }}{{P_{L} + P}}$	0.96258
D-R	$V = V_{0} \exp [ - D\ln^{2} (P^{0} /P)]$	0.97209
T	$V = \frac{{K_{b} P^{{}} V_{L} }}{{\left[ {1 + \left( {K_{b} P} \right)^{m} } \right]^{\frac{1}{m}} }}$	0.98384
E-L	$V = \frac{{K_{b} P^{{}} V_{L} }}{{1 + K_{b} P + m\sqrt[{}]{{K_{b} P}}^{{}} }}$	0.98866
F	$V = K_{b} P^{m}$	0.93715
D-A	$V = V_{0} \exp \left[ { - D\ln^{m} \left( {P^{0} /P} \right)} \right]$	0.99581
Quadratic	$V = aP^{2} + bP^{{}} + c$	0.95887
Exponential	$V = a\exp \left( {bP} \right) + c$	0.95883

P_L_—Langmuir pressure, MPa; V_L_—Langmuir volume, cm^3^/g; V_m_—monolayer maximum adsorption capacity, cm^3^/g; P^0^—saturated vapor pressure, MPa; C—adsorption heat constant; D-R—Dubinin-Astakhov model; V_0_—maximum adsorption capacity, cm^3^/g; k—Henry constant; L—Langmuir model; D—net adsorption heat constant; B-BET—Two-parameter Brunauer–Emmett–Teller model; D-P—Dual-Porosity model;T—Toth model; E-L—Extended Langmuir model; F—Freundlich model; D-A—Dubinin-Astakhov model.

**Table 3:** Optimization of adsorbed gas model in desorption.

Model	Model expression	Adsorption process *R*^*2*^
Weibull	$V = V_{0} [1 - \exp ( - bp^{q} )]$	0.99485
L-d	$V = \frac{{ab_{m} p}}{{1 + b_{m} p}} + c$	0.97720
D-A-d	$V = V_{0} \exp \left[ { - D\ln^{m} \left( {P^{0} /P} \right)} \right] + d$	0.99731
L-F-d	$V = \frac{{K_{b} P^{m} V_{L} }}{{1 + K_{b} P^{m} }} + d$	0.99649
D-R-d	$V = V_{0} \exp \left[ { - D\ln^{2} \left( {P^{0} /P} \right)} \right] + d$	0.99235
D-P-d	$V = kP + \frac{{PV_{L} }}{{P_{L} + P}} + d$	0.99376
Logarithm	$V = a\ln \left( P \right) + d$	0.84981
Power	$V = aP^{b} + d$	0.97938
Quadratic	$V = aP^{2} + bP^{{}} + d$	0.97770

b_m_—Combined function of adsorption heat, MPa^-1^; a—a constant of fit; b—a constant of fit; d—Residual adsorption capacity, cm^3^/g; V_0_—Maximum adsorption capacity, cm^3^/g; k— Henry's constantt ; q—adsorbate occupancy ratio; P_L_—Langmuir pressure, MPa; V_L_—Langmuir volume, cm^3^/g; P^0^—saturated vapor pressure, MPa; K_b_—Empirical constant, related to the adsorbent.

**Figure 5:**
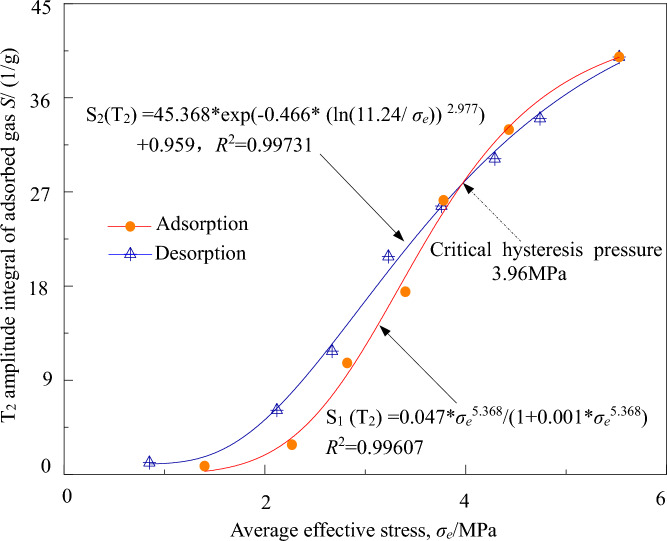
Relationships between micro *T*_*2*_ amplitude integral of adsorbed gas and the average effective stress.

The relationship between the adsorbed gas amount (integral of adsorbed gas *T*_2_ spectrum amplitude) and the average effective stress during the adsorption–desorption process can be obtained from Tables 3 and 4. The highest degree of fitting is achieved using the L-F model (R_2_ = 0.99609) and the D-A-d function model (*R*_2_ = 0.99731). Therefore, the L-F model and the D-A-d function model are selected as the optimal representation models for adsorbed gas in the coal-beaing shale gas adsorption–desorption process, respectively. The relationship between the adsorbed gas amount *S*(*T*_2_) per unit mass (1/g) during the adsorption–desorption process and the average effective stress *σ*_e_ is shown in Fig. 5.

**Table 4:** The content of coal-bearing shale gas in adsorption.

Average effective stress/MPa	1.40	2.27	2.82	3.40	3.78	4.43	5.53
Gas content/(cm^3^/g)	0.67	1.68	2.46	2.89	3.31	3.65	3.98

The quantity of adsorbed gas and the average effective stress(σ_e_) in the adsorption process conforms to the Langmuir–Freundlich (L-F) function model. According to Fig. 5, the fitting curve for *S*_1_(*T*_2_) is S_1_(*T*_2_) = 0.047 * σ_e_^5.368^ / (1 + 0.001 * σ_e_^5.368^), with an R-squared value (*R*_2_) of 0.99607. As the average effective stress increases, the adsorption space and matrix adsorption surface area of coal-bearing shale increase, leading to a gradual increase in the adsorbed gas quantity. The adsorption rate initially increases and then decreases.

(3) The desorption process shows a correlation between the desorbed gas quantity(*S*_2_(*T*_2_)) in coal-bearing shale and the average effective stress (σ_e_), which follows the Dubinin-Astakhov-Deo (D-A-d) model. According to Fig. 5, the fitting curve for *S*_2_(*T*_2_) is *S*_2_(*T*_2_) = 45.368 * exp(− 0.466 * (ln(11.24/σ_e_))^2.977^) + 0.959, with an R-squared value (*R*_2_) of 0.99731. As the average effective stress decreases, the adsorbed gas quantity in coal-bearing shale gradually decreases, while the desorbed gas quantity increases. Within the range of 1.40 to 3.96 MPa for the average effective stress, the desorption process in coal-bearing shale exhibits a lag compared to the adsorption process, with a critical lag pressure of 3.96 MPa.

#### Porous medium-confined gas quantity and the average effective stress conforms to linear function model

**Figure 6:**
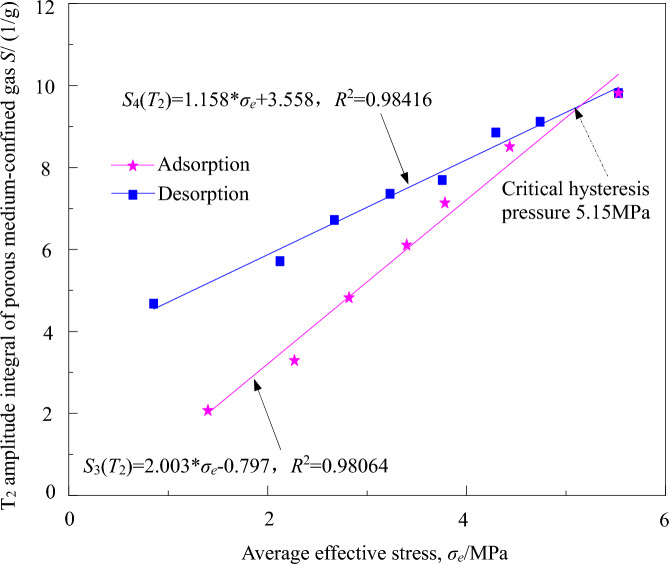
Relationships between micro *T*_*2*_ spectrum amplitude integral of porous medium-confined gas and the average effective stress.

The porous medium-confined gas in coal-bearing shale can be quantitatively characterized by integrating the amplitude of the micro-porous medium-confined gas *T*_2_ spectrum. As observed from the scatter plot of the integrated amplitude of the micro-porous medium-confined gas *T*_*2*_ spectrum and the average effective stress during the adsorption–desorption process, it is evident that the porous medium-confined gas in coal-bearing shale per unit mass (integrated amplitude of the porous medium-confined gas *T*_2_ spectrum) (*S*(*T*_2_)) and the average effective stress (σ_e_) exhibit a clear linear relationship. The fitted curve is shown in Fig. 6.

(1) During the adsorption process, the quantity of porous medium-confined gas follows a linear function model with the average effective stress.

From Fig. 6, it can be seen that during the "adsorption" process, the porous medium-confined gas quantity in coal-bearing shale follows a linear function model with the average effective stress. The fitted curve is *S*_3_(*T*_2_) = 2.003 * σ_e_—0.797, with *R*^2^ = 0.98064. As the average effective stress increases, the porous medium-confined gas quantity in coal-bearing shale exhibits a linear increase trend.

(2) During the desorption process, the porous medium-confined gas quantity follows a linear function model with the average effective stress.

From Fig. 6, it can be observed that during the "desorption" process, the porous medium-confined gas quantity in coal-bearing shale follows a linear function model with the average effective stress. The fitted curve is *S*_4_(*T*_2_) = 1.158 * σ_e_ + 3.558, with *R*^2^ = 0.98416. As the average effective stress decreases, the porous medium-confined gas quantity in coal-bearing shale shows a linear decrease trend. The porous medium-confined gas desorption in coal-bearing shale gradually increases. Within the range of average effective stress of 1.40–5.15 MPa, the desorption process of porous medium-confined gas exhibits hysteresis, with a critical hysteresis pressure of 5.15 MPa.

### Adsorption–desorption laws and quantitative characterization of the gas in coal-bearing shale

The quantity of coal-bearing shale gas under three-dimensional stress state can be approximately quantitatively characterized by the sum of the integral of the microscopic adsorbed and porous medium-confined gas *T*_2_ spectrum amplitudes (The influence of gas in the solid solution state and the “space” micro-trapped gas in the nuclear magnetic resonance probe is not taken into account.).

#### The quantity of coal-bearing shale gas and the average effective stress conform to the L-F and D-A-d models, respectively

**Figure 7:**
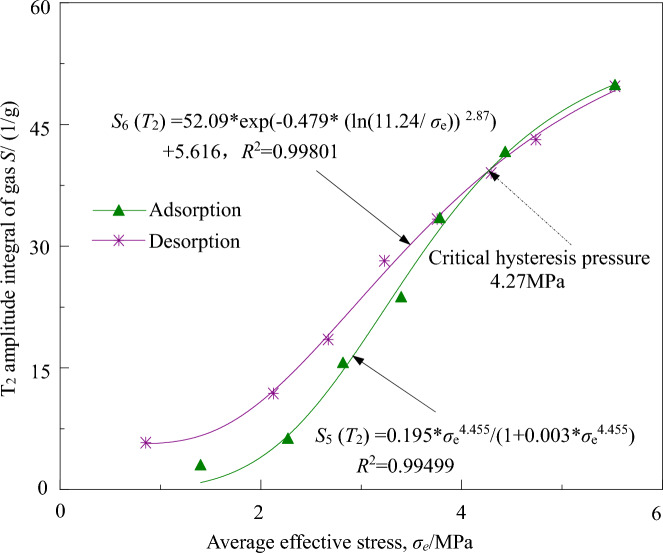
Relationships between gas micro *T*_*2*_ spectrum amplitude integral of coal-bearing shale gas and the average effective stress.

From Figs. 3 and 4, it can be observed that the nuclear magnetic resonance signal of the microscopic adsorbed gas in coal-bearing shale is much greater than that of the porous medium-confined gas, indicating that the gas mainly exists in the form of microscopic adsorption in experimental coal-bearing shale gas. Here, an attempt is made to fit the relationship between the quantity of coal-bearing shale gas (the sum of the integral of the microscopic adsorbed and porous medium-confined gas *T*_2_ spectrum amplitudes) per unit mass (1/g), and the average effective stress using the optimal model for adsorption–desorption of microscopic adsorbed gas. From the results shown in Fig. 7, it can be observed that there is a good fitting performance.

(1) The adsorption process of coal-bearing shale gas quantity conforms to the L-F function model with average effective stress. From Fig. 7, the fitting curve *S*_5_ (*T*_2_) for the adsorption process of coal-bearing shale gas quantity with the average effective stress is given by:

$$S_{{5}} \left( {T_{{2}} } \right) \, = \, 0.{195 }* \, \sigma_{{\text{e}}}^{{{4}.{455}}} / \, \left( {{1 } + \, 0.00{3 }* \, \sigma_{{\text{e}}}^{{{4}.{455}}} } \right),R^{{2}} = \, 0.{99499}.$$

As the average effective stress increases, the coal-bearing shale gas quantity gradually increases. When the average effective stress increases successively from 1.40 MPa to 2.27, 2.82, 3.40, 3.78, 4.43, and 5.53 MPa, the average effective stress increases by 62.20%, 24.15%, 20.54%, 11.31%, 17.24%, and 24.67% ,respectively. Correspondingly, the coal-bearing shale gas quantity increases by 115.40%, 152.74%, 52.44%, 41.44%, 24.41%, and 19.88% respectively. As the average effective stress increases, the adsorption quantity of coal-bearing shale gas under three-dimensional stress state gradually increases.

(2) The desorption process of coal-bearing shale gas quantity conforms to the D-A-d function model with the average effective stress. From Fig. 7, the fitting curve *S*_6_ (*T*_2_) for the desorption process of coal-bearing shale gas quantity with the average effective stress is given by:

$$S_{{6}} \left( {T_{{2}} } \right) \, = { 52}.0{9 }*{\text{ exp}}\left( { - 0.{479 }* \, \left( {{\text{ln}}\left( {{11}.{24}/\sigma_{{\text{e}}} } \right)} \right)^{{{2}.{87}}} } \right) \, + { 5}.{616},R^{{2}} = \, 0.{998}0{1}.$$

As the average effective stress decreases, the coal-bearing shale gas quantity gradually decreases, and the desorption quantity of coal-bearing shale gas gradually increases. In the range of average effective stress from 1.40 to 4.27 MPa, the desorption process of coal-bearing shale gas exhibits hysteresis relative to the adsorption process, with a critical hysteresis pressure of 4.27 MPa.

#### Macroscopic characterization of coal-bearing shale gas conforms to the logarithmic function model

In the adsorption–desorption process of coal-bearing shale gas under three-dimensional stress state, the effective volume of the gas experimental chamber (L-NMR core holder cavity) is 8.81 cm^3^, and the effective volume of the gas reference chamber in the constant-temperature water bath is 27.36 cm^3^. Using the calculation method for reference coal and shale isothermal adsorption quantity, the macroscopic gas quantity at various pressure equilibrium points during the adsorption–desorption process of coal-bearing shale gas under three-dimensional stress state is shown in Tables 4 and 5.

**Table 5:** The content of coal-bearing shale gas in desorption.

Average effective stress/MPa	5.53	4.74	4.29	3.76	3.23	2.67	2.12	0.85
Gas content/(cm^3^/g)	3.98	3.70	3.59	3.21	2.81	2.56	2.10	0.49

**Figure 8:**
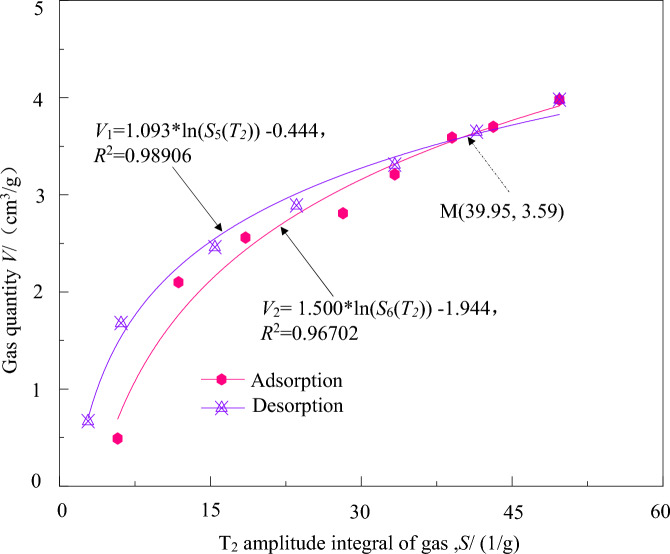
Relationships between gas quantity and the *T*_*2*_ spectrum integral amplitudes of coal-bearing shale gas.

Analysis of the data in Fig. 7 and Tables 4 and 5 reveals that the adsorption–desorption process of coal-bearing shale gas under three-dimensional stress state exhibits a logarithmic function relationship between the macroscopic gas content of the coal-bearing shale gas and the integrated value of its microscopic *T*_2_ spectral amplitude. This relationship is depicted in Fig. 8.

The fitting curve for the relationship between macroscopic gas content and *T*_2_ spectral amplitude integral of microscopic gas during the adsorption process is *V*_1_ = 1.093*ln(*S*_5_(*T*_2_))−0.444, *R*^2^ = 0.98906. During the adsorption process, the macroscopic gas content in coal-bearing shale gradually increases in a logarithmic function form with an increasingly smaller amplitude as the micro-nuclear magnetic resonance signal increases.

The fitting curve for the relationship between macroscopic gas content and *T*_2_ spectral amplitude integral of microscopic gas during the desorption process is *V*_2_ = 1.500*ln(*S*_6_(*T*_2_))−1.944, *R*^2^ = 0.96702. During the desorption process, the macroscopic gas content in coal-bearing shale gradually decreases in a logarithmic function form with an increasingly larger amplitude as the micro-nuclear magnetic resonance signal decreases.

The correlation curve between the macroscopic and microscopic gas content in coal-bearing shale gas during the adsorption–desorption process follows a logarithmic function model, but the change patterns are not consistent. There is a critical value of 39.95 (dimensionless), which corresponds to an approximate average effective stress of 3.59 MPa. The differences in the correlation curves between macroscopic and microscopic gas content in coal-bearing shale gas during the adsorption–desorption process may be related to the stress field of the coal-bearing shale gas reservoir.

Based on the quantitative characterization curves of macroscopic and microscopic gas content in coal-bearing shale gas during the adsorption–desorption process as shown in Fig. 8, the macroscopic gas content of coal-bearing shale gas under corresponding operating conditions can be quickly determined through the integration of L-NMR *T*_2_ spectral amplitude. This enables real-time online monitoring of the adsorption–desorption quantity of coal-bearing shale gas under complex stress state.

## Conclusion

The experiments on the adsorption–desorption characteristics of coal-bearing shale gas under three-dimensional stress state using L-NMR spectrum have yielded the following conclusions:

1. The *T*_2_ spectra of coal-bearing shale gas adsorption–desorption under three-dimensional stress state exhibit a tri-peak characteristic.
2. During the adsorption process, the adsorption quantity of coal-bearing shale gas can be modeled using the L-F function model in relation to the average effective stress, while the desorption process follows the D-A-d function model.
3. Both the adsorption and desorption processes for coal-bearing shale gas under three-dimensional stress state are characterized by logarithmic function models. The differences in quantitative characterization curves may be related to the elastic–plastic deformation, damage and fracture of coal-bearing shale micro-pores, as well as the hysteresis of pore gas desorption and the stress field in the existing state.

## Data Availability

The datasets generated and analysed during the current study are not publicly available due [Research on the next stage is still ongoing and will not be made public for the time being] but are available from the corresponding author on reasonable request.
